# Commentary: The Case for Abandoning Therapeutic Chelation of Copper Ions in Alzheimer’s Disease

**DOI:** 10.3389/fneur.2017.00503

**Published:** 2017-09-25

**Authors:** Rosanna Squitti, Carlo Salustri, Mauro Rongioletti, Mariacristina Siotto

**Affiliations:** ^1^Molecular Markers Laboratory, IRCCS Istituto Centro San Giovanni di Dio—Fatebenefratelli, Brescia, Italy; ^2^Institute of Cognitive Sciences and Technologies (CNR), Rome, Italy; ^3^Department of Laboratory Medicine, Research and Development Division, San Giovanni Calibita—Fatebenefratelli Hospital, Rome, Italy; ^4^Don Carlo Gnocchi ONLUS Foundation, Milan, Italy

**Keywords:** copper, Alzheimer’s disease, beta-amyloid peptide, chelation therapy, metals

Drew’s paper (1) contains a helpful overview of the numerous works published on the interaction between copper and beta-amyloid (Aβ) in Alzheimer’s disease (AD). However, his linking of the usefulness of therapeutic copper chelation in AD primarily to copper–Aβ coordination chemistry appears to us to be limited. First, Aβ’s “causative” role is challenged by various types of evidence; second, one must consider that AD is a complex disease, contributing to the development of many risk factors.

While still trusting pathogenesis theories, such as the “amyloid cascade” (2), or the “metal hypothesis” (3), and more recent ones proposing a loss of Aβ’s capability to properly handle copper (4–6), one should not overlook the literature on the relationship between AD stages and copper concentrations in serum, plasma, cerebrospinal fluid, and the brain. Many studies have investigated whether a significant difference in copper levels exists between AD patients and healthy individuals, and whether those levels correlate with the patient clinical picture.

Drew notes the controversies surrounding the conclusions of these studies. Nevertheless, a number of meta-analyses published in the last 5 years undoubtedly show that the copper levels are lower in AD brains than in healthy ones [see, e.g., the comparison of 115 AD patients with 123 healthy controls by Ref. (7)], while the copper levels are higher in AD patients’ serum [see, e.g., Wang et al.’s comparison of 1,768 AD patients with 2,514 healthy controls (8)]. Subsequent studies have shown the similar results. Three studies confirmed lower levels of copper in the brain (9–11). Considering general circulation studies, two studies reported no overall differences in outcomes, while five studies show higher levels of copper in AD patients vs. controls (Table 1). Moreover, a recent meta-analysis (12) comparing 879 AD patients with 1712 controls has shown that the plasma copper component that circulates not bound to ceruloplasmin (non-Cp copper) is increased in AD, as confirmed by studies published after this publication (13, 14).

**Table 1:** Studies on copper in Alzheimer’s disease (AD) patients and healthy controls [published after the meta-analysis published in 2015 (8)].

Study (year of publication)	Country	AD patients (*n*)	Healthy controls (*n*)	AD patients, ^a^plasma/serum copper (mean ± SD)	Healthy controls, ^a^plasma/serum copper (mean ± SD)	Statistical significance	Trend	Notes
Talwar et al. (14)	India	108	105	22.1 ± 9.6 μmol/L	18.7 ± 7 μmol/L	*p*-value = 0.003	↑	Age group, sex, and education adjusted
Guan et al. (15)	China	92	116	1.52 ± 0.33 mg/L; 23.92 ± 5.2 μmol/L	1.29 ± 0.284 mg/L; 20.3 ± 4.5 μmol/L	*p*-value < 0.001	↑	
Paglia et al. (16)	Italy	34	40	703.88 ± 244.03 μg/L; 11.079 ± 3.84 μmol/L	815.75 ± 206 μg/L; 12.83 ± 3.24 μmol/L	Not significant	No change	Twenty-four subjects with subjective memory complaint (SMC; 858.96 ± 224.15 μg/L) had increased copper than healthy controls (*p*-value = 0.049)
Pu et al. (17)	China	125	40	20.31 ± 6.74 (μmol/L)	16.32 ± 6.34 (μmol/L)	*p*-value = 0.037	↑	Controls vs. moderate 42 AD
Vaz et al. (18)	Brazil	32	32	Median 0.060^b^ mg/L (0.01 P75–P25 interquartile range); 0.94 µmol/L	Median 0.048^b^ (0.007 P75–P25 interquartile range) mg/L; 0.94 µmol/L	*p*-value < 0.001	↑	^b^Copper was assessed in erythrocytes; data were median (P75–P25 interquartile range)
Koc et al. (19)	Turkey	45	33	0.9 (0.4–1.3) μg/mL, median (min–max); 14.2 (6.3–20.5) μmol/L	1.01 (0.5–1.5) μg/mL, median (min–max); 15.9 (7.9–23.6) μmol/L	0.1	No change	
Siotto et al. (20)	Italy	58	84	14.23 (2.3) μmol/L	15.46 (3.3) μmol/L	*p*-value < 0.001	↑	

*^a^Data are reported as the original values published and converted in µmol/L*.

*^b^The study of Vaz et al. (18) was carried out in erythrocytes and not in serum/plasma*.

Thus, AD appears as characterized by an aberrant copper homeostasis: higher non-Cp copper levels in the blood stream and lower levels in the brain, exactly like in Wilson disease (WD) (21), although at a much milder level. In fact, James and coworkers (22) demonstrated that in the brain areas strongly damaged by AD, lower levels of total copper coexist with higher levels of copper not bound to proteins (non-Cp copper in the brain), as it occurs in WD. Furthermore, lower values of serum ceruloplasmin specific activity coexist with higher levels of non-Cp copper (20). In summary, a mild degree of failure of copper control appears to occur in AD.

Recently, some genetic studies [reported in Ref. (23)] have contributed to our understanding by demonstrating that the presence of *ATP7B* (WD) gene’s variants increases (from 1.63 to 5.16 odds ratio) the risk of developing AD. The gene’s variant *K832R* has been the object of a recent *in-vivo* study demonstrating that this AD-related *ATP7B* variant is a loss-of-function allele in *Drosophila* (24). Further genetic studies are in progress to verify whether AD patients with higher non-Cp copper levels are also carriers of multiple rare *ATP7B* gene mutations. However, should that be the case, copper toxicosis could be possibly prevented/treated with chelating or anti-copper agents, as in WD.

Moreover, Brewer has drawn attention on possible beneficial effects of more tolerable zinc-based therapies. He (25) describes *post-hoc* results presented by Dr. Diana Pollack at various meetings, showing zinc therapy as capable of stopping cognitive decline: by restricting Pollack’s *post-hoc* analysis to those among her patients who were aged 70 years or older (14 zinc treated and 15 placebo treated), Brewer showed that the latter scored significantly better in the Alzheimer’s Disease Assessment Scale cognitive subscale, Clinical Dementia Rating Scale Sum of Boxes, and Mini–Mental State Examination than placebo patients.

In 2002, our laboratory attempted a study on the effects of d-penicillamine (d-pen) in AD (26). Penicillamine is known to promote “de-coppering” in WD by forming copper–penicillamine complexes, which are then excreted with the urine. In our study, 77.8% of the AD patients under d-pen treatment showed a 24-h urinary excretion higher than 200 μg/day, which is 5× the value (40 μg/day) which is widely accepted as representative of a “normal” copper excretion (27). A 5 μg/day × 40 μg/day excretion is considered the “d-pen challenge test” cutoff, supportive of WD diagnosis in asymptomatic patients (28). The d-pen test, however, is not disease-specific. Individuals suffering from hepatic cirrhosis, for example, also show excretion values higher than the mentioned cutoff. In AD patients under d-pen treatment (26), this is suggestive of failure a copper control.

Unfortunately, when excretion reaches levels that make the copper balance negative, some patients treated with WD under d-pen treatment show a serious “iatrogenic” deterioration (often referred to as “paradoxical effect”), with an increase of the neurological symptoms. This effect is believed to be caused by a frantic mobilization and redistribution of copper which results in high copper level in the brain and in the blood (29, 30). Manifestation of similar neurological symptoms in our study on AD forced an early interruption (26). This is suggestive, however, of copper homeostasis abnormalities, as some authors commented (31). Nevertheless, up to that point, d-pen had shown a positive effect in reducing oxidative stress.

In conclusion, we agree with Drew that *the metal–A*β*_1–x_interactions* can be *downstream of an underlying pathology* of AD. However, we deem this not to be a valid reason for *abandoning therapeutic chelation of copper ions in AD* that has not been yet thoroughly tested. In our opinion, anti-copper therapies appear to have excellent chances to affect positively the condition for a percentage of AD patients, in agreement with the notion of the existence of different AD “sub-types” (32–35).

## Author Contributions

RS, CS, MR, and MS contributed to the concepts expressed in the commentary. All authors contributed to the conceptualization of the manuscript, its realization, and writing. In particular, MS evaluated the chemical coordination part of the Drew’s manuscript and the clinical chemistry part of our commentary. RS reviewed the meta-analysis section, and MR and CS reviewed the genetic part. CS revised English editing.

## Conflict of Interest Statement

RS reports personal fees in the past 3 years and other interests from Canox4drug SPA (Italy), and IGEA Research Corporation (Miami, FL, USA) outside the submitted work. Other authors have no conflict of interest to declare.
